# Investigation of Surface Micro-Mechanical Properties of Various Asphalt Binders Using AFM

**DOI:** 10.3390/ma15124358

**Published:** 2022-06-20

**Authors:** Yueqin Hou, Yun Chen, Haiwei Zou, Xiaoping Ji, Dongye Shao, Zhengming Zhang, Ye Chen

**Affiliations:** 1 School of Human Settlements and Civil Engineering, Xi’an Jiaotong University, Xi’an 710049, China; houyueqin527@163.com; 2 Key Laboratory for Special Area Highway Engineering of Ministry of Education, Chang’an University, Xi’an 710064, China; jixp@chd.edu.cn (X.J.); 2021121177@chd.edu.cn (Y.C.); 3 Shenzhen Municipal Design & Research Institute Co., Ltd., Shenzhen 518029, China; kuaire92@163.com; 4 Highway Engineering Management Office, Xi’an 710065, China; wchenye2@163.com (D.S.); wscysnm@163.com (Z.Z.)

**Keywords:** asphalt binders, atomic force microscope, elastic modulus, nanohardness, aging, water, anti-stripping agent

## Abstract

The microstructure of asphalt affects the micro-mechanical properties. In this study, atomic force microscopy (AFM) was used to investigate the surface elastic modulus and nanohardness of asphalt binder. Relevant mechanical indexes were quantitatively evaluated by contact mechanical model. Five types of asphalts, including different grades, oil sources, and before and after modification, were selected as test objects, and the effects of asphalt binder type, aging, water, and anti-stripping agent on the asphalt micromechanics were explored. The results showed that the micromechanical properties of asphalt binder are affected by grade, oil source, and modification. The aging resistance of modified asphalt binder is better than that of unmodified asphalt binder. Water immersion reduces the surface micromechanical properties of the asphalt binder. The effect of the anti-stripping agent on the modified asphalt binder is greater than that of the unmodified asphalt binder.

## 1. Introduction

Asphalt binder is a viscous liquid or semi-solid material after petroleum under reduced pressure distillation which is widely used as the adhesive material for asphalt pavement [1]. Asphalt binder is neither a uniform nor unidirectional system but contains a microstructure of a few to a tenth of micrometers, containing about 105~106 different chemicals, which is a material with complex structure and composition [2]. Asphalt pavement has the advantages of smooth surface and comfortable driving. As one of the main materials of asphalt pavement, asphalt binder affects the durability of asphalt pavement. With the development of material mechanics, the research has gradually developed from macroscale to micro scale [3]. The researchers hope to explore the properties of asphalt binders at the micro scale and provide a theoretical basis for the further improvement and optimization of asphalt binders in the future.

Atomic Force Microscopy (AFM) is an instrument that provides surface morphology, nanohardness, microscopic modulus, and viscosity at the microscopic level of a material, and has advantages of simple sample preparation and controllable environment. Related studies show that AFM could test the micromorphology and mechanical properties of asphalt binder at the molecular scale, and the test results are unique and reproducible, which is one of the powerful means to study the microscopic properties of asphalt binder [4,5,6]. Nazzal et al. studied the special bee-like structure of asphalt by AFM and explored the effect of modifiers [7]. Nivedya et al., studied the change of microstructure before and after immersion [8]. Relevant information is extracted from the force–displacement curve, and the nanohardness, friction force, and elastic modulus of the material can be obtained [9]. Adriana Garcia proposed an analytical method for the microscopic performance of asphalt binders [10]. Liu explored the modulus and adhesion of asphalt binder by AFM [11]. Chen et al., tested the micromechanical properties of the aggregates and investigated the effect of the chemical composition of the aggregates on the mechanical properties [12]. Zhu et al., found that AFM can accurately measure the hardness of materials [13]. Tarefder RA et al., investigated the micromechanical properties of asphalt binder and asphalt concrete [14]. With the further development of the research, the researchers found that the microstructure of asphalt binder directly affects its mechanical properties, such as hardness, elastic plasticity, adhesion force, surface energy, and healing behavior [15,16]. Rashid et al. tested the stiffness, microstructure, and modulus of asphalt binder through AFM, and found that modulus and stiffness were associated with the asphalt microstructure [17]. Ji et al. tested the microstructure and surface energy of asphalt and aggregates based on AFM and explored the relationship between asphalt binder surface energy and microstructure [18,19,20]. Some scholars have also studied the asphalt binder aging behavior, destructive behavior, and adhesion behavior through AFM. Pei and Xie et al., tested the nanohardness and elastic modulus of asphalt binders before and after aging and revealed the hardening behavior of asphalt binder aging from the micro scale [21,22]. Mansourkhaki et al., comprehensively evaluated the influence mechanism of the regenerator on asphalt binder adhesion and recommended the optimal amount of the regenerator [23]. Allen R G et al., studied the effect of asphalt chemical composition on the structure and microscopic properties of asphalt binder [24]. Tarefder RA et al., explored the causes and influencing factors of moisture damage to asphalt binders using AFM [25].

To date, most of the related research has focused on the surface micromorphology and special bee structure of asphalt binder, and further research on surface micromechanical properties is still needed to investigate. The micromechanical performance of asphalt binder surface is affected by type, aging, moisture, and modifier, and the factors cross each other. However, the micromechanical properties of asphalt binders in various states are rarely studied.

The objective of this paper is to study the changes in micromechanical properties (elastic modulus and nanohardness) of asphalt binders with different grades, oil sources, and before and after modification under different states (original state, aging, water immersion, anti-stripping agent).

To address the research gap, this paper first used AFM to obtain force curves for different regions of asphalt binder and calculated the elastic modulus and nanohardness combined with contact mechanics theory. Next, the asphalt binder was treated with aging, water immersion, and anti-stripping agent to explore the micro-mechanical behavior. The above micro method has laid a foundation for the improvement and optimization of asphalt binder.

## 2. Materials

Five types of asphalt binders were selected as test objects, including different grades (SK-70, SK-90), different oil sources (SK-70, KL-70, Shell-70), and whether they were modified (Shell-70 and modified-Shell). The modified-Shell asphalt was prepared by adding 4% SBS modifier to Shell-70 by high speed shear. The properties of the tested asphalt binder were shown in Table 1.

## 3. Methodology

### 3.1. Testing Method

#### 3.1.1. Asphalt Binder Preparation in Different States

The preparation of asphalt with aging, water immersion, and addition of anti-stripping agent were as follows:

(1) Aging. The asphalt binder was aged at 163 °C for 85 min using a rotating film oven, and then taken out for sample preparation.
(2) Immersion in water. Put the prepared asphalt sample into a vessel filled with water, and the water level submerges the asphalt sample, soak it for 24 h, take it out, and let it dry for testing.
(3) Anti-stripping agent. The asphalt was heated to 165 °C, an anti-stripping agent of 0.5% of the asphalt mass was added, and the samples were prepared after stirring uniformly.

#### 3.1.2. Sample Preparation

**Figure 1:**
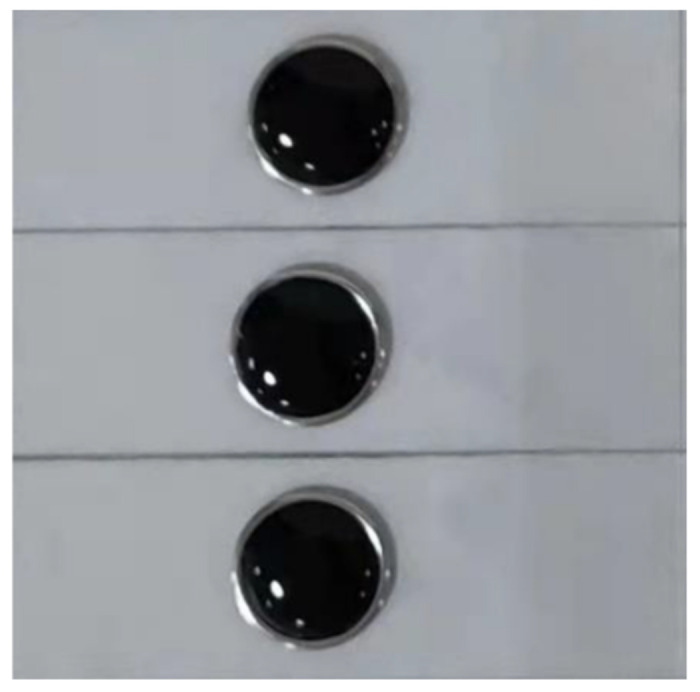
Asphalt samples.

In order for the asphalt binder to meet the test requirements, it was chosen to be naturally solidified by dropping it into a ring mold at high temperature, and three samples of each asphalt binder were prepared for parallel testing. The samples are shown in Figure 1. The asphalt binder samples were prepared in the following steps.

Step 1: Place the rectangular mica sheet (18 mm × 13 mm × 0.1 mm) on the slide and place the ring mold (10 mm outer diameter, 8 mm inner diameter) on the rectangular mica sheet.
Step 2: Heat the asphalt binder 150–160 °C, naturally drop the liquid asphalt to the ring mold, and then put it into the 150–160 °C oven for 20 min to smooth the sample surface.
Step 3: Remove the asphalt binder sample into airtight plastic trays for airtight storage.

#### 3.1.3. Force Curve Test

**Figure 2:**
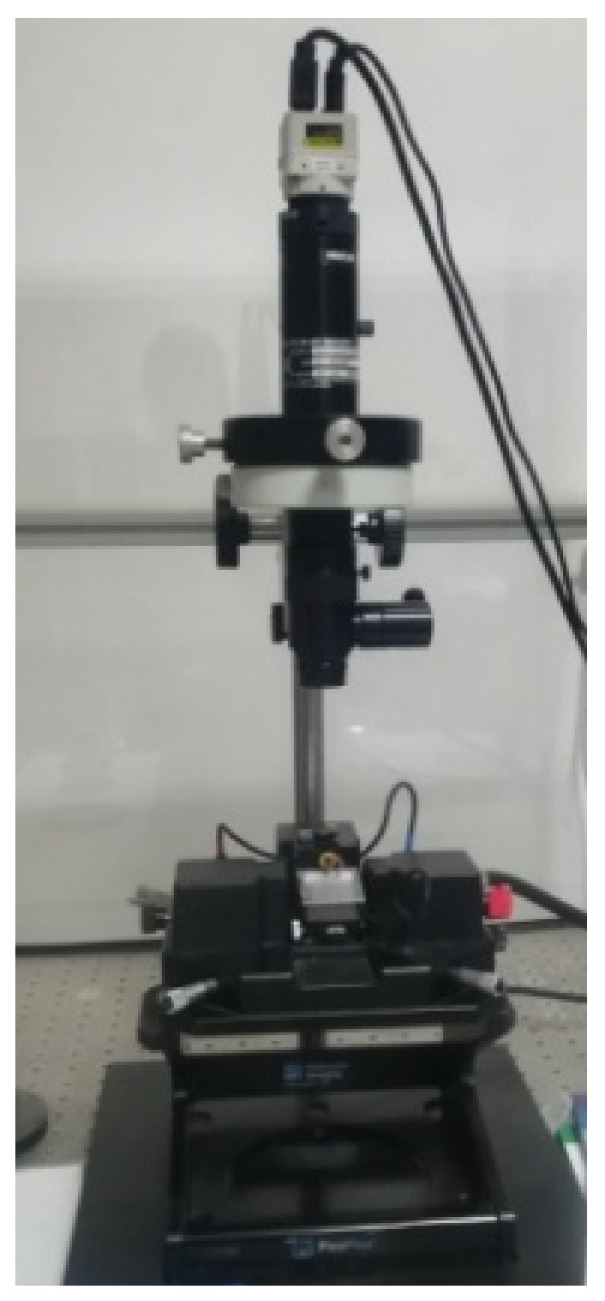
AFM (agilent5400).

The nacreous surface of asphalt binder is not homogeneous and presents a partially special structure (bee-type structure). A related study shows that the micromechanical properties of asphalt binder surface are related to the special structure [19]. Therefore, this paper first tested the surface micromorphology of asphalt binder, then tested the force curves of different regions, and finally performed regional statistical calculations on asphalt. The agilent 5400 atomic force microscopy was used for the test, as shown in Figure 2. The testing process included microform scanning and force curve testing, and a silicon nitride probe with a radius of curvature of 7 nm and a K value of 7.4 was selected. The test steps are as follows.

Step 1: Place the asphalt binder sample on the AFM carrier table, set the scan area and frequency, and conduct a sensitivity test for the probe.
Step 2: In tapping mode, scan the surface micro-topography of the asphalt binder sample, and mark the peak, valley, and other flat areas of asphalt binder.
Step 3: Switch to the contact mode to obtain the force curves of marked the peak, valley, and other flat areas.

**Figure 3:**
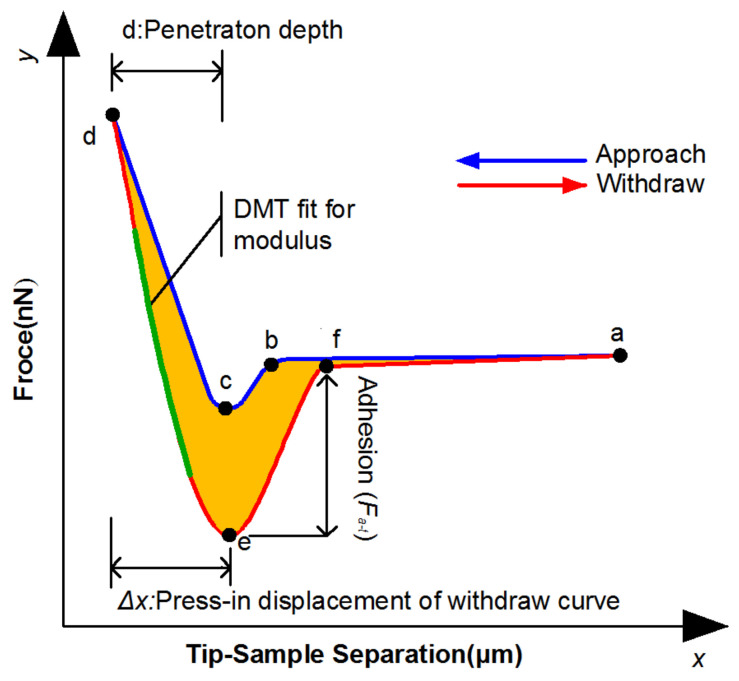
Force curve schematic diagram.

Figure 3 shows a schematic diagram of the force curves, a-b is the section where the probe is gradually approaching the surface of the sample, sections b-c are the probes jumping up and contacting the sample surface under the action of surface tension, sections c-d are the elastic deformation of the sample caused by the probe continuing to press into, section d-e is for the probe to start to retreat, sections e-f show that the probe did not separate from the sample surface under the action of adhesion, section f-a is the probe detached from the sample surface.

By extracting and calculating the information from the force curve, the surface micromechanical properties of the test sample were obtained. 

### 3.2. Micro-Mechanical Properties

After extracting the force curve information, the surface micromechanical parameters of asphalt binder, including surface micro-elastic modulus and nano-hardness, can be calculated with the contact mechanics model [26,27], and the area-weighted average was calculated as the test result by combining the asphalt binder test area and force curve. The effects of aging, water immersion, and anti-stripping agent on the micro-mechanical properties of asphalt binder were analyzed.

#### 3.2.1. Surface Micromechanics Indicators

(1) Elastic modulus *E*

Elastic modulus is one of the important performance parameters of engineering materials. When a force is applied to an elastic material, in the elastic deformation stage, its stress and strain become proportional, and the proportionality coefficient is called elastic modulus.

The probe is in contact with the asphalt binder surface, and a small force is applied to the asphalt to ensure that the deformation produced by the asphalt binder is within the elastic range. This contact is similar to the direct interaction between a rigid blob and an elastomer, and the DMT model (Derjaguin–Muller–Toporov) in contact mechanics is used to fit the analysis of the probe retraction process.

The relationship between the load and the indentation depth is shown in Equation (1). Knowing the micro-force *F_t_* exerted by the probe, the adhesion force *F_a_* between the probe and asphalt binder, the curvature radius *R* of the probe and the deformation amount *δ* of the asphalt binder, the composite elastic modulus E* of asphalt binder and probe can be obtained by inverse calculation through Equation (1).

(1) $$F_{t}-F_{a}=\frac{4}{3}E^{*}\sqrt{R}\delta^{\frac{3}{2}}$$

The composite elastic modulus E* of the asphalt binder and the probe is shown in Equation (2). Knowing the Poisson’s ratio and the elastic modulus of the probe (*υ_p_* and *E_p_*), the Poisson’s ratio of the asphalt binder (*υ_a_*) is 0.25 [28]. The microscopic elastic modulus of the asphalt binder can be obtained by inverse calculation.

(2) $$E^{*}=\left[\frac{1-\upsilon_{s}^{2}}{E_{s}}+\frac{1-\upsilon_{t}^{2}}{E_{t}}\right]$$

(2) Surface nanohardness *H*

Knowing the maximum test load *P_max_*, composite elastic modulus E*, the correction coefficient *β*, the penetration depth *d*, and the contact area *A* of probe and asphalt, the nanohardness *H* of asphalt binder can be obtained, as shown in Equations (3) and (4).

(3) $$H=\frac{P_{\max}}{25.4h_{c}^{2}}$$

(4) $$d_{c}=d-\frac{\varepsilon P_{\max}}{2\beta E^{*}}\sqrt{\frac{\pi }{4}}$$

#### 3.2.2. Area-Weighted Average of Surface Micromechanics Indicators

**Figure 4:**
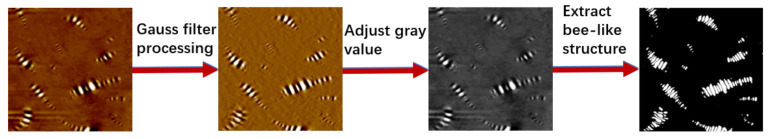
Image Processing of Bee-like Structure.

The microstructure of the asphalt binder within a certain area (20 × 20 µm^2^) was observed by AFM, then the graph was pre-processed in the AFM data analysis software Nanoscope, and the area was calculated using a Matlab program, as shown in Figure 4.

**Figure 5:**
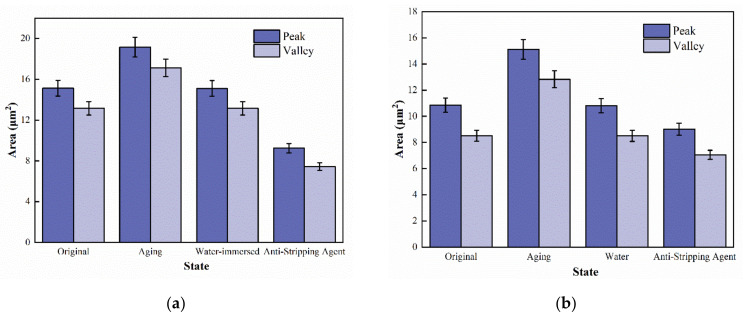

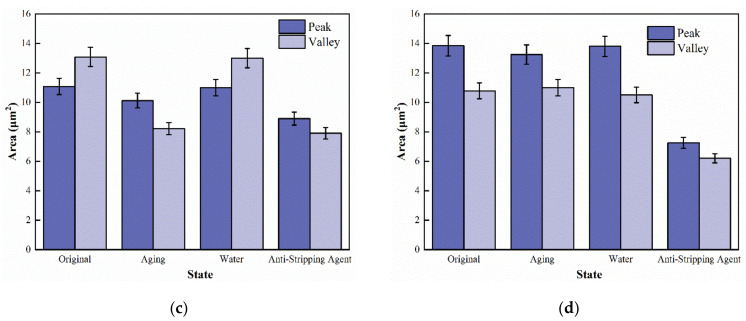
Statistical map of different regions of asphalt binder micro-morphology: (**a**) SK-70, (**b**) Shell-70, (**c**) SK-90, and (**d**) Modified-Shell.

Figure 5 shows the statistical results of each area of asphalt binder in different states, in which KL-70 asphalt binder has no bee-shaped structure and no statistical data.

Nine force curves were obtained for each asphalt, including 3 force curves for the peak area, the valley area, and other areas. The area-weighted average was calculated based on the asphalt binder test area and the force curves of different areas as the test result.

(5) $$X=\frac{\sum P_{i}\times X_{i}}{400}$$

where *X* is the weighted average of the area of the surface micromechanical properties, *X_i_* is the surface micromechanical properties of different regions, *P_i_* is the area of the different areas.

## 4. Results and Discussion

### 4.1. Influence of Asphalt Binder Type

**Figure 6:**
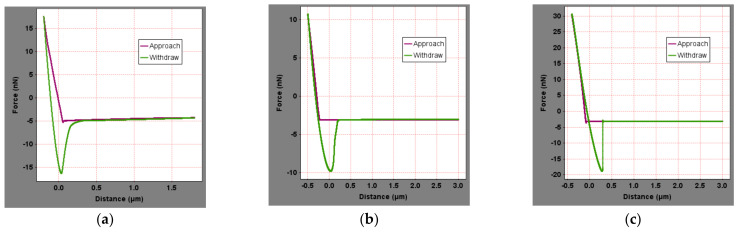

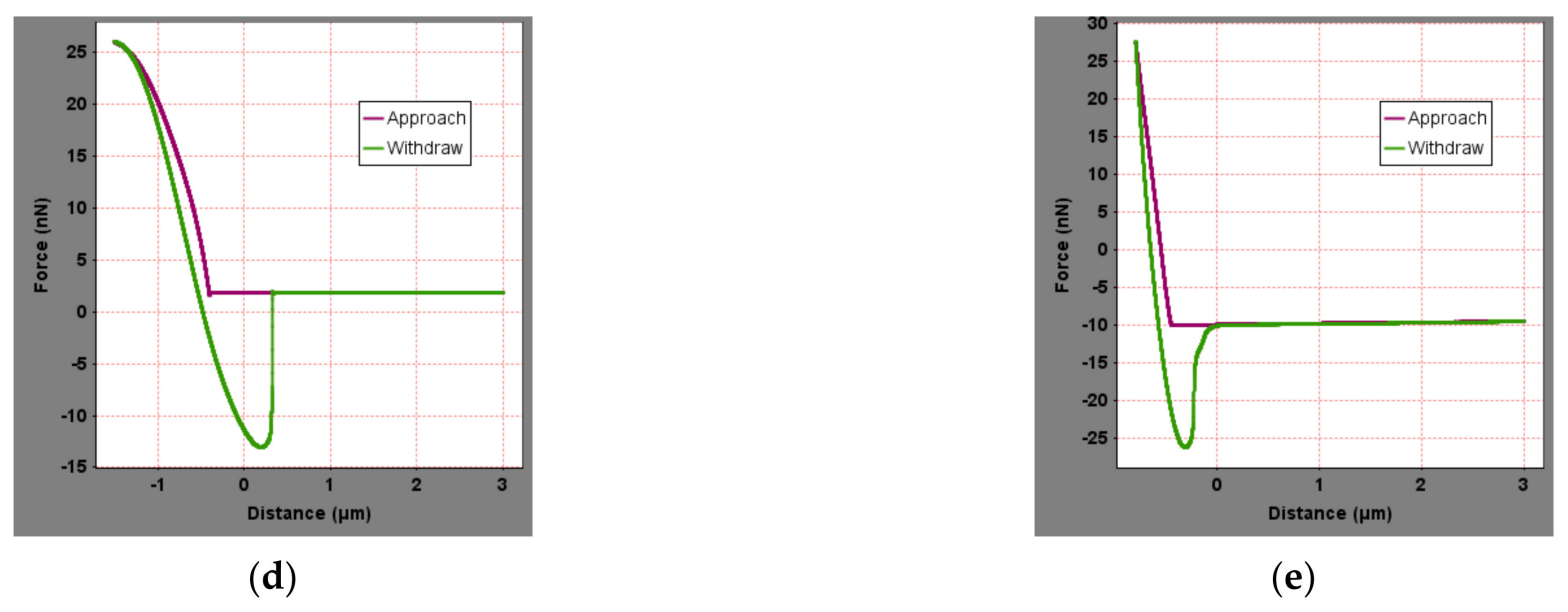
Force curves of original asphalt binder: (**a**) SK-70, (**b**) KL-70, (**c**) Shell-70, (**d**) SK-90, and (**e**) Modified−Shell.

**Figure 7:**
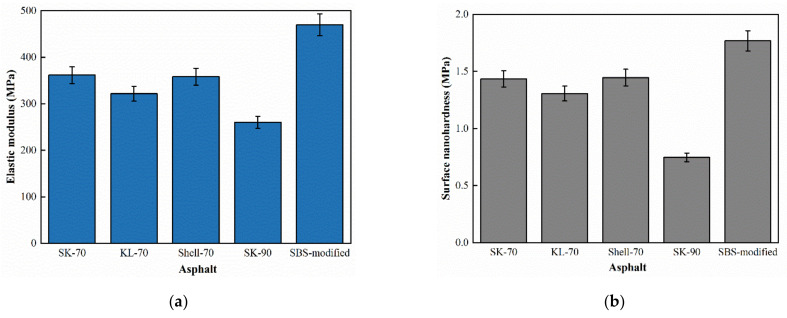
Surface micromechanical properties of original asphalt binder: (**a**) Elastic modulus and (**b**) Surface nanohardness.

To calculate the surface micromechanical properties of asphalt binder, the force curve of five asphalt binders in different areas were obtained. Figure 6 showed the distance-force curves of five asphalt binders in peak area in original state. To save space, the force curves of other regions are not shown in this paper. Extracting the force curve information and the surface micromechanical properties of the asphalt binder, including elastic modulus and nanohardness, can be calculated from Equations (1)–(4), as shown in Figure 7.

Surface microscopic nanohardness and elastic modulus both reflect the deformation resistance of asphalt binder [22]. In this paper, nanohardness and elastic modulus of asphalt binders have a similar pattern as SK-90 < KL-70 < SK-70 < Shell-70 < modified-Shell. As the asphalt binder grade increases, the softer the asphalt binder surface, the lower the nano-hardness, rather the opposite, the higher the elastic modulus. The elastic modulus and nanohardness of the modified asphalt binder are greater than those of unmodified asphalt binder due to the block copolymer in the SBS modifier that increases the physical crosslinking region in the asphalt binder, which improved the deformation resistance of asphalt binder and increases the surface hardness of the asphalt binder [29].

### 4.2. Influence of Aging

**Figure 8:**
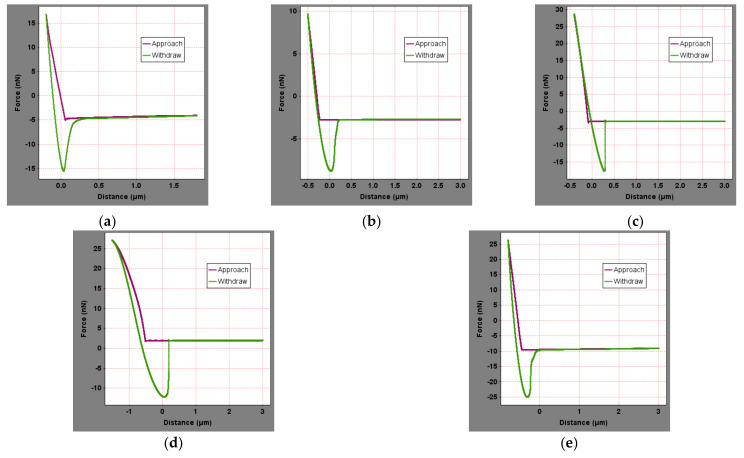
Force curves of aged asphalt binder: (**a**) SK-70, (**b**) KL-70, (**c**) Shell-70, (**d**) SK-90, and (**e**) Modified−Shell.

**Figure 9:**
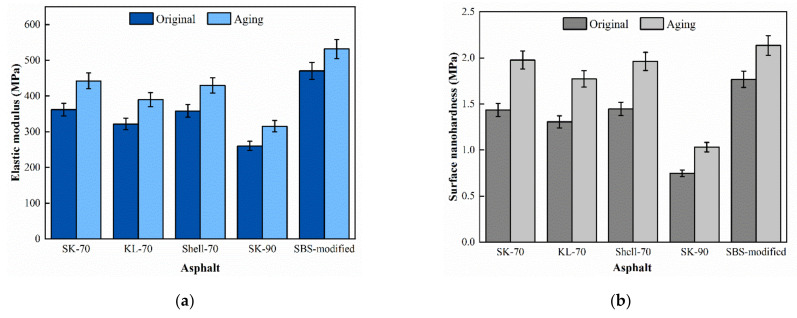
Surface micromechanical properties of aged asphalt binder: (**a**) Elastic modulus and (**b**) Surface nanohardness.

The force curves of the aged asphalt binder were shown in Figure 8, and the calculated micromechanical properties of asphalt binder were compared with the properties in the original state, as shown in Figure 9. After aging, the asphalt binder showed an increase in elastic modulus and nanohardness, the modulus of five asphalt binders increased by 22.28%, 21.15%, 21.17%, 20.01%, and 13.05%, respectively, and the nano-hardness increased by 37.82%, 38.04%, 35.66%, 35.71%, and 20.83%, sequentially. This indicates that the asphalt binder specimens tend to become “hard” and “brittle” after aging. The growth rate of the modified asphalt binder is lower than that of the unmodified, indicating that the SBS modifier can improve the anti-aging performance of asphalt binder. Combined with related studies, a mesh structure which was formed by modifier and asphalt binder avoided the rapid volatilization of small molecules during the aging process [30].

### 4.3. Influence of Water

**Figure 10:**
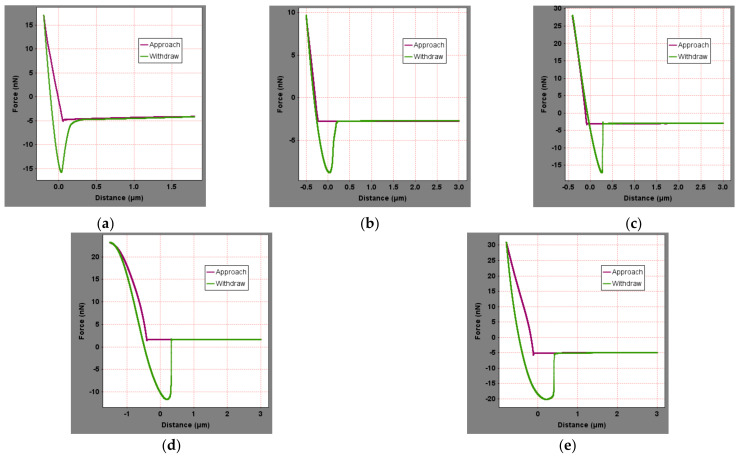
Force curves of asphalt binder after water-immersed: (**a**) SK-70, (**b**) KL-70, (**c**) Shell-70, (**d**) SK-90, and (**e**) Modified−Shell.

**Figure 11:**
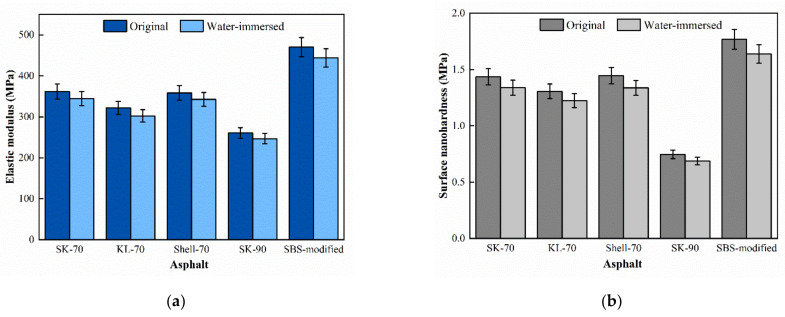
Surface micromechanical properties of asphalt binder after water-immersed: (**a**) Elastic modulus and (**b**) Surface nanohardness.

The force curves of the asphalt binder after water immersion were shown in Figure 10, and the surface microscopic mechanical properties of asphalt binder were calculated for comparison with those in the original state, as shown in Figure 11. After water immersion, the microscopic elastic modulus and nano-hardness of the asphalt binder showed a small decrease, where the elastic modulus decreased by 4–6% and the nanohardness decreased by 6–8%, which may be due to the puncture of the asphalt binder film resulting in a small decrease in the surface nano-hardness. The reason for this phenomenon could be that moisture damaged part of the surface asphalt film, exposing the part under the asphalt film and causing a decrease in the overall elastic modulus and nanohardness [31].

### 4.4. Influence of Anti-Stripping Agent

**Figure 12:**
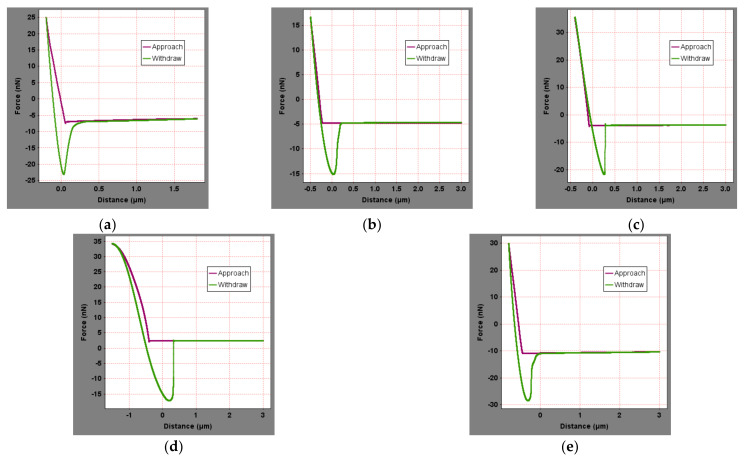
Force curves of asphalt binder doped with anti-stripping agent: (**a**) SK-70, (**b**) KL-70, (**c**) Shell-70, (**d**) SK-90, and (**e**) Modified−Shell.

**Figure 13:**
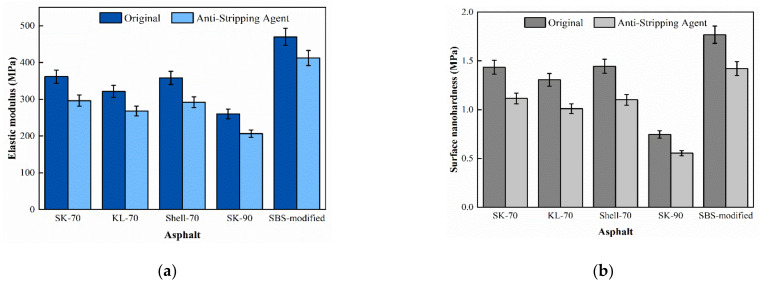
Surface micromechanical properties of asphalt doped with anti-stripping agent: (**a**) Elastic modulus and (**b**) Surface nanohardness.

The force curves of the asphalt binder with the anti-stripping agent (ATA) were shown in Figure 12, and the calculated surface micromechanical properties of the asphalt binder are compared with those in the original state, as shown in Figure 13. After the addition of ATA, the elastic modulus of five asphalt binders were decreased by 18.03%, 20.67%, 16.51%, 18.36%, and 12.24%, sequentially. The modified asphalt binder decreased less than the unmodified asphalt binder. The ATA reduced the ability of asphalt binder to resist deformation, which is due to the fact that ATA increased the lightweight component of asphalt binder, the intermolecular forces are weakened, the molecular motility is correspondingly increased, and the resistance to deformation becomes weaker [32].

After incorporation of anti-stripping agent, the nanohardness of five asphalt binders were decreased by 22.24%, 25.78%, 22.65%, 23.82%, and 19.56%, sequentially. This is for ATA increasing the lightweight component of the asphalt binder, which reduces the surface hardness of the asphalt binder.

## 5. Conclusions

In this paper, the surface micromechanical properties of asphalt binder were investigated by AFM, including elastic modulus and nanohardness. The effects of asphalt type, aging, water immersion, and anti-stripping agent on the surface micromechanics of asphalt binder were investigated. The main conclusions were as follows:

(1) Micromechanical properties of asphalt binder were affected by grade, oil source and modification. Micromechanical properties decreased with the increase of asphalt grade (SK-90 < SK-70). The oil source has an effect on micromechanical properties of the asphalt binder (SK-70 < KL-70 < Shell-70). Micromechanical properties of the modified asphalt were better than those of unmodified (Shell-70 < modified-Shell).
(2) The aging resistance of modified asphalt binder was better than that of unmodified. The aging behavior increased the elastic modulus and nanohardness of the unmodified asphalt binder by 20.01–22.28% and 35.66–38.04%, respectively, and increased that of the modified asphalt binder by 13.05% and 20.83%, respectively.
(3) Water immersion reduces the micromechanical properties of asphalt binder. After being immersed in water, the elastic modulus of the five asphalt binders decreased by 4–6%, and the nanohardness decreased by 6–8%.
(4) The effect of the anti-stripping agent on the modified asphalt binder is greater than that of the unmodified asphalt binder. After adding the anti-stripping agent, the elastic modulus and nanohardness of the unmodified asphalt binder decreased by 16.51–20.67% and 22.24–25.78%, respectively, and that of the modified asphalt binder decreased by 12.24% and 19.56%, respectively.

## 6. Suggestions for Further Research

The AFM on-site detection temperature is room temperature (25 ± 2 °C), which does not match the on-site temperature of the road surface. How to correlate the test temperature with the on-site actual temperature will be further solved in future research.

It is recommended to further combine the DSR test to explore the relationship between the macro-mechanical index and the micro-performance of asphalt binder.

It is recommended to further explore the changes of the four components and their functional groups of the asphalt binder under different conditions, and to further study the water damage, aging, and the action mechanism of the anti-stripping agent of the asphalt binder.

## Figures and Tables

**Table 1:** Performance Index of Asphalt.

Asphalt	Penetration (0.1 mm)	Ductility (cm)	Softening Point (°C)	After RTFOT		
				Penetration Ratio (%)	Ductility (cm)	Mass Loss (%)
SK-70	80	77.0	50.1	71.4	7.5	−0.28
KL-70	69	84.5	46.1	67.4	7.5	−0.31
Shell-70	78	42.5	50.1	62.5	7.4	−0.50
SK-90	94	110.3	47.6	60.5	8.8	−0.79
Modified-Shell	72	78.5	77.1	83.8	26.3	−0.48

## References

[1] Wang C., Wang M., Chen Q., Zhang L. (2022). Basic performance and asphalt smoke absorption effect of environment-friendly asphalt to improve pavement construction environment. J. Clean. Prod..

[2] Ji X., Hou Y., Zou H., Chen B., Jiang Y. (2020). Study of surface microscopic properties of asphalt based on atomic force microscopy. Constr. Build. Mater..

[3] Gong X. (2017). Multi-Scale Domain Mechanical Behavior and Unified Model of Asphalt Pavement Materials.

[4] Rebelo L., de Sousa J., Abreu A., Baroni M.P.M.A., Alencar A., Soares S., Filho J.M., Soares J. (2014). Aging of asphaltic binders investigated with atomic force microscopy. Fuel.

[5] Lv P. (2013). Using Atomic Force Microscope to Study the Effect of Sample Thickness on Fracture Energy of Asphalt.

[6] Bellitto V. (2012). Atomic Force Microscopy: Imaging, Measuring and Manipulating Surfaces at the Atomic Scale.

[7] Nazzal M.D., Abu-Qtaish L., Kaya S., Powers D. (2015). Using Atomic Force Microscopy to Evaluate the Nanostructure and Nanomechanics of Warm Mix Asphalt. J. Mater. Civ. Eng..

[8] Nivedya M.K., Trottier T.G., Yu X., Tao M., Burnham N.A., Mallick R.B. (2019). Microstructural Evolution of Asphalt Binder under Combined Action of Water and Pressure. J. Transp. Eng. Part B Pavements.

[9] Fu M., Wang H.C., Hong Y.S. (2000). Micro/nano-scale material mechanical properties testing. Prog. Mech..

[10] García A., Aguiar-Moya J.P., Salazar-Delgado J., Baldi-Sevilla A., Loría-Salazar L.G. (2019). Methodology for estimating the modulus of elasticity of bitumen under different aging conditions by AFM. Road Mater. Pavement Des..

[11] Liping L. (2018). A Method of Determination of Micro Scale Properties of Asphalt Components in Mixtures Based on Atomic Force Microscopy. J. Tongji Univ. Nat. Sci..

[12] Chen Y., Hou Y., Ji X., Zou H., Dai C., Chen B. (2021). Characterization of surface mechanical properties of various aggregates from micro scale using AFM. Constr. Build. Mater..

[13] Zhu L.-N., Xu B.-S., Wang H.-D., Wang C.-B. (2014). Comparison of Four Different Methods to Determine the Hardness of Plasma-sprayed Cr3C2–NiCr Coating by Nano-indentation. J. Test. Eval..

[14] Tarefder R.A., Zaman A.M., Uddin W. (2010). Determining hardness and elastic modulus of asphalt by nanoindentation. Int. J. Geomech..

[15] Loeber L., Muller G., Morel J., Sutton O. (1998). Bitumen in colloid science: A chemical, structural and rheological approach. Fuel.

[16] Lesueur D., Gerard J.F., Claudy P., Letoffe J.M., Planche J.P., Martin D. (1996). A structure-related model to describe asphalt linear viscoelasticity. J. Rheol..

[17] Rashid F., Hossain Z., Bhasin A. (2019). Nanomechanistic properties of reclaimed asphalt pavement modified asphalt binders using an atomic force microscope. Int. J. Pavement Eng..

[18] Ji X., Li J., Zou H., Hou Y., Chen B., Jiang Y. (2020). Multi scale investigation on the failure mechanism of adhesion between asphalt and aggregate caused by aging. Constr. Build. Mater..

[19] Ji X., Chen Y., Hou Y., Dai C., Chen B., Zou H. (2021). Surface microscopic properties of various aggregates using laser scanning confocal microscope. Constr. Build. Mater..

[20] Ji X., Sun E., Zou H., Hou Y., Chen B. (2020). Study on the multiscale adhesive properties between asphalt and aggregate. Constr. Build. Mater..

[21] Pei Z.S. (2016). Analysis of AFM-based microscopic characteristics and influencing factors of aging asphalt surface. Ph.D. Thesis.

[22] Xie S. (2017). Research on Nanostructure and Adhesion Characteristics of Asphalt Surface in Room Temperature Domain. Ph.D. Thesis.

[23] Mansourkhaki A.A.M. (2019). Application of different modifiers for improvement of chemical characterization and physical-rheological parameters of reclaimed asphalt binder. Constr. Build. Mater..

[24] Allen R.G., Little D.N., Bhasin A., Glover C.J. (2014). The effects of chemical composition on asphalt microstructure and their association to pavement performance. Int. J. Pavement Eng..

[25] Tarefder R.A., Zaman A.M. (2010). Nanoscale evaluation of moisture damage in polymer modified asphalts. J. Mater. Civ. Eng..

[26] Pang X. (2015). Asphalt and Aggregate Adhesion Characteristics Analysis Based on the Principle of AFM and the Surface Energy. Ph.D. Thesis.

[27] Oliver W.C., Pharr G.M. (1992). An improved technique for determining hardness and elastic modulus using load and displacement sensing indentation experiments. J. Mater. Res..

[28] Zhang T., Yang Y. (2002). Development and application of nano-hardness technology. Adv. Mech..

[29] Li J. (2019). Study on Microscopic Properties of SBS Modified Asphalt before and after Aging Based on AFM.

[30] Wang M., Liu L. (2017). Investigation of microscale aging behavior of asphalt binders using atomic force microscopy. Constr. Build. Mater..

[31] Gopalakrishnan K., Birgisson B., Taylor P., Attoh-Okine N.O. (2011). Nanotechnology in Civil Infrastructure: A Paradigm Shift.

[32] Shen A., Wang J., Guo Y., Zhou X., Zhou T. (2019). Study on the effect of anti-spalling agents on the aging performance of asphalt. Highway.

